# Characteristics and long-term outcomes of childhood glaucoma: a retrospective-cohort study

**DOI:** 10.12688/f1000research.51256.1

**Published:** 2021-03-01

**Authors:** Supawan Surukrattanaskul, Pukkapol Suvannachart, Sunee Chansangpetch, Anita Manassakorn, Visanee Tantisevi, Prin Rojanapongpun

**Affiliations:** 1 Queen Sirikit National Institute of Child Health, Bangkok, Thailand; 2 Glaucoma Research Unit, Faculty of Medicine, Chulalongkorn University and King Chulalongkorn Memorial Hospital, Bangkok, Thailand

**Keywords:** childhood glaucoma, congenital glaucoma, paediatric glaucoma, Childhood Glaucoma Research Network classification, retrospective cohort, paediatric eye disease

## Abstract

**Purpose**: To evaluate the clinical characteristics and treatment outcomes of patients with childhood glaucoma.

**Methods**: We retrospectively reviewed the data of patients with childhood glaucoma who visited the glaucoma clinics at the Queen Sirikit National Institute of Child Health and the King Chulalongkorn Memorial Hospital between January 2008 and January 2018. The diagnosis was based on the Childhood Glaucoma Research Network classification. We recorded their clinical characteristics and requirement of any glaucoma interventions.

**Results**: A total of 691 eyes from 423 patients were included in this study. The patients predominantly comprised boys. The average follow-up duration was 71.3±63.8 months. The mean age at presentation was 3.9±4.4 years. Most patients presented with a high initial intraocular pressure (IOP) of 28.5±11.2 mmHg. Glaucoma associated with non-acquired ocular anomalies (22.9%) was the most common subtype, followed by primary congenital glaucoma (20.8%). We recorded a family history of glaucoma in 6.4% of patients. Most patients had bilateral glaucoma (63.4%) and required at least one intervention (51.5%). The average IOP at the latest follow-up visit was 19.1±10.8 mmHg. All glaucoma types had significantly lower IOP, compared to that at their baselines (all p<0.001). Moreover, most patients had an unfavourable visual acuity (49.5%) at their latest visit.

**Conclusions**: Secondary glaucoma associated with non-acquired ocular anomalies is the most common subtype of glaucoma. All subtypes, including primary glaucoma, were sporadic. The majority of patients had unfavourable visual outcomes. These real-world findings are fundamental to acquire a better understanding of childhood glaucoma.

## Introduction

Childhood glaucoma is a vision-threatening disorder with an incidence of 2.29 to 5.41 per 100,000 individuals ^1, 2^. The diagnosis of childhood glaucoma poses some challenges. This can be attributed to the variation in clinical presentations among different age groups. Despite high intraocular pressure (IOP) being the primary cause of glaucomatous damage, an accurate IOP measurement is not always obtained in children. Angle surgery is a common therapy but it is mostly associated with unfavourable outcomes in children, compared to that in adults ^3^.

Childhood glaucoma encompasses several categories of glaucoma. The Childhood Glaucoma Research Network (CGRN) classification was proposed by an international consortium of glaucoma specialists in 2013 to standardise the definition of childhood glaucoma subtypes ^4^. The prevalence of childhood glaucoma differs among ethnicities, ranging from 1:1,250 to 1:68,254 live births ^5– 12^. The incidence and clinical characteristics of childhood glaucoma in Thailand have not yet been reported. The Queen Sirikit National Institute of Child Health is one of the largest tertiary centres in Thailand. It is responsible for the treatment of a majority of the complex paediatric cases from all over the country. Paediatric glaucoma clinics have been established by the joint collaboration between the Queen Sirikit National Institute of Child Health and the King Chulalongkorn Memorial Hospital, a university-based hospital. These clinics aimed to treat all paediatric glaucoma cases that were referred to the aforementioned hospitals and have been operated by the same group of glaucoma specialists for more than 10 years. We aimed to describe the clinical characteristics and brief long-term treatment outcomes of the large paediatric glaucoma cohorts of the two major referral centres in Thailand.

## Methods

### Cohort selection

We retrospectively reviewed the medical records of all patients who had been examined at the paediatric glaucoma clinics of the Queen Sirikit National Institute of Child Health and the King Chulalongkorn Memorial Hospital between January 2008 and January 2018. The patient list was extracted from the hospital database to include all individuals that had at least one visit to the paediatric glaucoma clinic during the above period and/or subjects that had the ICD-10-CM diagnostic codes of Q15.0 and all H40 and H42 categories. The inclusion criteria were patients who aged <16 years at the time of the first clinic visit and met the CGRN glaucoma or glaucoma suspect definition ^4^. Cases with incomplete medical record precluding the diagnosis were excluded. The CGRN definition of glaucoma and suspected glaucoma has been previously described ^4^.

### Ethics approval

Ethical approval was obtained from the Research Ethic Committee of the Queen Sirikit National Institute of Child Health and Faculty of Medicine, Chulalongkorn University (REC.041/2562 and IRB.807/61). The requirement for written informed consent was waived due to the retrospective nature of the study.

### Outcomes

We collected data for the demographic characteristics, initial clinical presentations, and diagnoses. All available clinical information was evaluated and classified according to the Childhood Glaucoma Research Network (CGRN) classification into the following seven groups: (1) primary congenital glaucoma (PCG), (2) juvenile open-angle glaucoma (JOAG), (3) secondary glaucoma following cataract surgery (SCG-C), (4) secondary glaucoma associated with non-acquired systemic disease or syndrome (SCG-S), (5) secondary glaucoma associated with non-acquired ocular anomalies (SCG-O); (6) secondary glaucoma associated with acquired conditions (SCG-A); and (7) glaucoma suspect (GS). The CGRN classification diagram has been illustrated elsewhere ^4, 13^.

We recorded the interventions during the follow-up course and final outcomes, including visual acuity (VA) and IOP at the latest available visit in eyes with a confirmed glaucoma diagnosis (diagnosis group 1 to 6). For glaucoma interventions, we reviewed the data to determine if the subjects had received any incisional surgeries (i.e. trabeculectomy, trabeculotomy, and glaucoma drainage device implantation), cyclodestructive laser procedures (i.e. diode transscleral cyclophotocoagulation, diode laser endoscopic cyclophotocoagulation), or a combination of both at any time point of the follow-up period.

The best-corrected VAs were determined using the LEA or Snellen chart at 10 feet or 20 feet, respectively. In contrast, VA was graded by the fixation patterns using a central, steady, and maintained (CSM) technique for patients who were too young to determine the pictures or numbers ^14^. The LEA chart symbols were reproduced with permission from Good-Lite Co., Elgin, IL. According to Karr *et al.* ^15^, the fixation pattern of CSM, CSUM, CUSUM and UCUSUM was estimated as the VA of ≥ 20/30, 20/30–20/100, ≤20/300, and ≤5/200, respectively. We extrapolated the Snellen acuity from the fixation grade with a modification from Karr *et al*.’s method and classified the VA into the following three groups: (1) favourable: best-corrected VA (BCVA) ≥20/70 or fixation grade of CSM, (2) moderately favourable: BCVA=20/70 to <20/400 or fixation grade of CSUM; and (3) unfavourable: BCVA≤20/400 or fixation grade of CUSUM or UCUSUM.

### Statistical analysis

The categorical data were presented as counts and percentages. We conducted the Shapiro-Wilk test for the normality of continuous data distribution. The data were reported as means and standard deviations or medians and interquartile ranges, depending on the distribution. We used the analysis of variance to compare the initial IOP among the glaucoma subtypes and the paired t-test to compare the IOP during the initial and latest visit. Furthermore, the Stuart-Maxwell test for marginal homogeneity was used to compare the proportion of matched pairs of the VA during the initial and latest visit. Statistical analyses were performed using Stata 13.0 (Stata Corp, College Station, TX, USA). A P-value <0.05 was deemed statistically significant.

## Results

The cohort comprised 423 patients (691 eyes). While 338 patients (532 eyes) were diagnosed with glaucoma, 85 patients (159 eyes) had GS. The average follow-up duration was 71.3±63.8 months (median 50; interquartile range, 22–112 months). Table 1 summarises the baseline characteristics. The average age at presentation was 3.91±4.40 years (median 1.75: interquartile range, 0.25–6.75 years). We recorded a family history of glaucoma in 15 (6.4%) patients. Furthermore, we found a statistically significant predominance of boys in all subjects (P=0.002), PCG (P=0.01), SCG-C (P=0.01), and GS (P=0.04). The mean of initial IOP was 28.5±11.2 mmHg. There was no difference in the initial IOP among the glaucoma subtypes (p=0.52). The most common presentation was cloudy eye (68.2%), which represented leukocoria or corneal haze, and megalocornea (14.5%). Table 2 demonstrates the mode of detection patterns and clinical presentations.

**Table 1.** Clinical and demographic characteristics of the study participants.

Characteristics	Total	PCG	JOAG	SCG-C	SCG-S	SCG-O	SCG-A	GS
Number of eyes (patients)	691 (423)	145 (88)	15 (8)	54 (33)	45 (32)	156 (97)	117 (80)	159 (85)
Gender (patients, %) Male Female	250 (59.1%) 173 (40.9%)	56 (63.6%) 32 (36.4%)	4 (50%) 4 (50%)	24 (72.7%) 9 (27.3%)	18 (56.3%) 14 (43.8%)	53 (54.6%) 44 (45.4%)	43 (53.8%) 37 (46.3%)	52 (61.2%) 33 (38.8%)
Age at presentation (years)	1.6 (0.3 to 6.8)	0.4 (0.2 to 4.4)	8.4 (5.3 to 12.4)	5.6 (1.8 to 7.6)	2.1 (0.4 to 6.3)	0.3 (0 to 3.0)	6.8 (2.8 to 10.4)	2.7 (0.6 to 7.7)
Family history (patients, %) * Yes No	15 (6.4%) 219 (93.6%)	0 35 (100%)	1 (20.0%) 4 (80.0%)	1 (7.7%) 12 (92.3%)	2 (13.3%) 13 (86.7%)	6 (10.7%) 50 (89.3%)	0 50 (100%)	5 (8.3%) 55 (91.7%)
Laterality (patients, %) Bilateral Unilateral	268 (63.4%) 155 (36.6%)	57 (64.8%) 31 (35.2%)	7 (87.5%) 1 (12.5%)	21 (63.6%) 12 (36.4%)	13 (40.6%) 19 (59.4%)	59 (60.8%) 38 (39.2%)	37 (46.3%) 43 (53.6%)	74 (87.1%) 11 (12.9%)
Refraction (eyes, SE) †	0 (-2.00 to 1.25)	2.13 (2.13 to 2.13)	0	-2.25 (-6.63 to 5.50)	0.50 (0 to 1.25)	-2.88 (-3.25 to 3.00)	-1.25 (-2.00 to -0.25)	0.63 (-0.50 to 1.50)
Corneal diameter (eyes, mm)	11.5 (10 to 13)	12.5 (12 to 13)	10.5 (10 to 12)	10 (10 to 12)	11.5 (9 to 12.3)	11 (9 to 12.3)	11 (10 to 12)	11 (10 to 12)
Cup to disc ratio (eyes) ‡	0.6 (0.3 to 0.8)	0.8 (0.6 to 0.9)	0.9 (0.9 to 1.0)	0.4 (0.3 to 0.7)	0.5 (0.3 to 0.8)	0.3 (0.2 to 0.4)	0.4 (0.3 to 0.7)	0.6 (0.5 to 0.8)

Data shown in median (interquartile range). * Data available in 234, 35, 5, 13, 15, 56, 50, and 60 patients for total, PCG, JOAG, SCG-C, SCG-S, SCG-O, SCG-A, and GS, respectively. † Data available in 93, 1, 2, 16, 5, 5, 19, and 45 eyes for total, PCG, JOAG, SCG-C, SCG-S, SCG-O, SCG-A, and GS, respectively. ‡ Data available in 426, 71, 14, 39, 34, 41, 71, and 156 eyes for total, PCG, JOAG, SCG-C, SCG-S, SCG-O, SCG-A, and GS, respectively. IOP, intraocular pressure; SE, spherical equivalence; PCG, primary congenital glaucoma; JOAG, juvenile open angle glaucoma; SCG-C, secondary glaucoma following cataract surgery; SCG-S, secondary glaucoma associated with non-acquired systemic condition; SCG-O, secondary glaucoma associated with ocular anomalies; SCG-A, secondary glaucoma associated with acquired conditions; GS, glaucoma suspect.

**Table 2.** Mode of detection patterns in eyes with a glaucoma diagnosis.

Mode of detection patterns	Total	PCG	JOAG	SCG-C	SCG-S	SCG-O	SCG-A
Presented with glaucoma-related symptoms Cloudy eye Megalocornea Epiphora Red eye Photophobia Blepharospasm Blurred vision	230 (69.1%) 49 (14.7%) 22 (6.6%) 14 (4.2%) 13 (3.9%) 2 (0.6%) 3 (0.9%)	68 (57.6%) 27 (22.9%) 13 (11.0%) 0 9 (7.6%) 1 (0.9%) 0	0 0 0 0 0 0 3 (100.0%)	7 (100.0%) 0 0 0 0 0 0	9 (40.9%) 10 (45.5%) 2 (9.1%) 1 (4.5%) 0 0 0	121 (85.8%) 10 (7.1%) 6 (4.3%) 4 (2.8%) 0 0 0	25 (59.5%) 2 (4.8%) 1 (2.4%) 9 (21.4%) 4 (9.5%) 1 (2.4%) 0
From clinical surveillance or screening (no glaucoma-specific symptoms) *	126	-	6	32	17	9	62
Unknown / missing data	73	27	6	15	6	6	13

Data shown in number of eyes (%). * Including eyes from clinical surveillance in known systemic or ocular diseases, clinical surveillance due to family history of glaucoma and other forms of child health screening. PCG, primary congenital glaucoma; JOAG, juvenile open angle glaucoma; SCG-C, secondary glaucoma following cataract surgery; SCG-S, secondary glaucoma associated with non-acquired systemic condition; SCG-O, secondary glaucoma associated with ocular anomalies; SCG-A, secondary glaucoma associated with acquired conditions.

Most patients had bilateral glaucoma (63.4%). Among the 155 eyes of the unilateral cases, there were 72 (46.5%) and 83 (53.6%) right and left eyes, respectively. The diagnosis with significantly higher bilateral presentation included PCG (P=0.01), JOAG (P=0.03), SCG-O (P=0.03), and GS (P<0.001). SCG-S (unilateral 59.4%) and SCG-A (unilateral 53.6%) comprised a higher proportion of unilateral cases. However, the difference was statistically insignificant.

**Figure 1.**
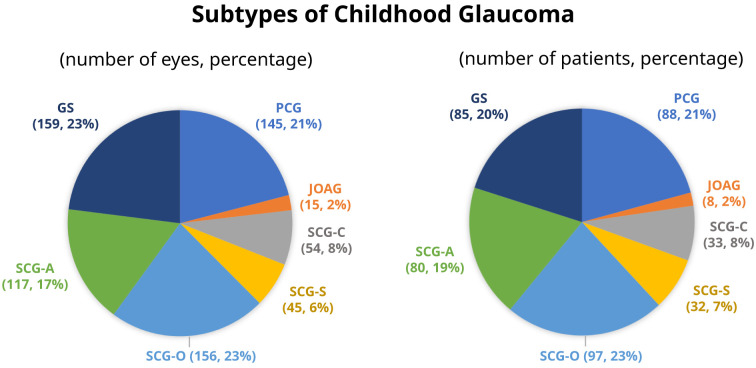
Subtypes of childhood glaucoma. PCG, primary congenital glaucoma; JOAG, juvenile open angle glaucoma; SCG-C, secondary glaucoma following cataract surgery; SCG-S, secondary glaucoma associated with non-acquired systemic condition; SCG-O, secondary glaucoma associated with ocular anomalies; SCG-A, secondary glaucoma associated with acquired conditions; GS, glaucoma suspect.

SCG-O was the most common subtype, accounting for 23% of the cohort or 29% of the glaucoma cases. In contrast, JOAG was the least common subtype. Figure 1 depicts the frequency of each glaucoma diagnosis.

**Figure 2.**
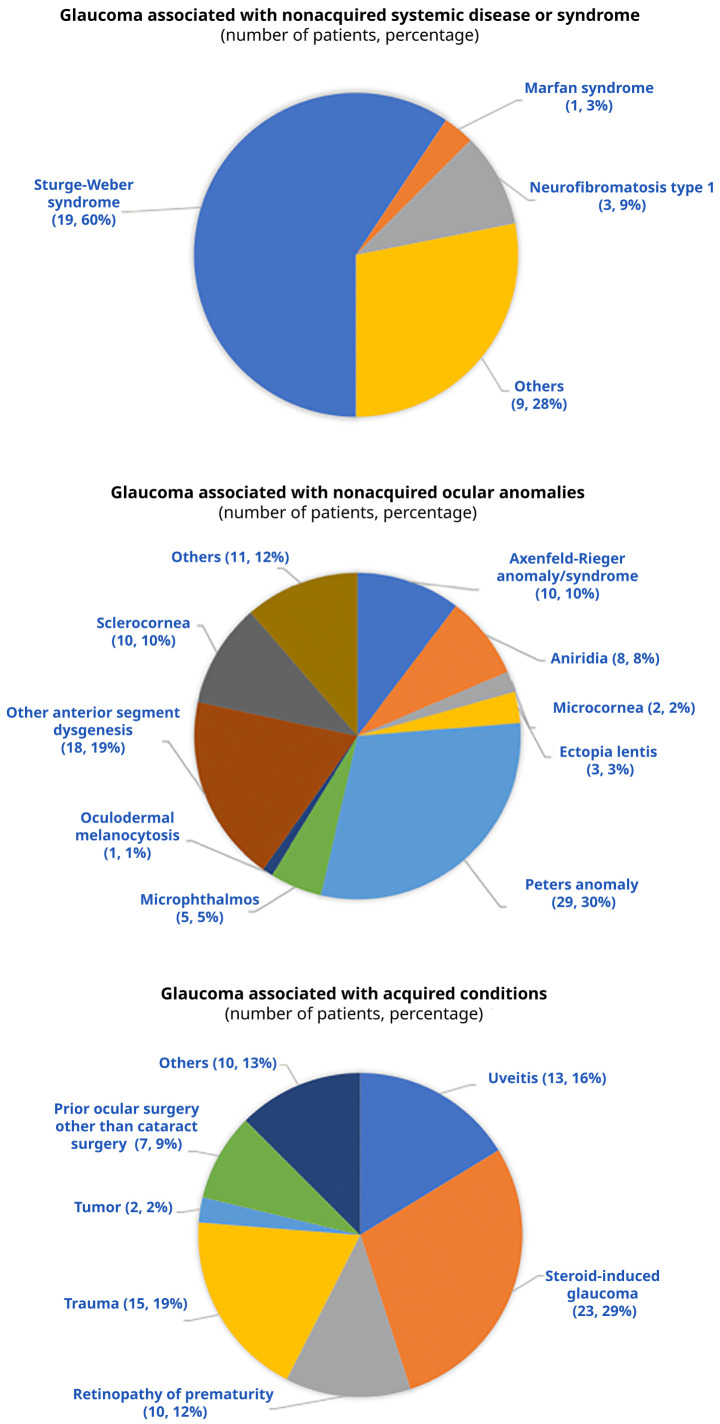
Causes of secondary glaucoma subtypes that are associated with non-acquired systemic disease, non-acquired ocular anomalies, and acquired conditions.

The onset of PCG was neonatal (≤1 month), infantile (>1 to 24 months), and late (>2 years) in 33 (37.5%), 38 (43.2%), and two (2.3%) patients, respectively. However, the onset was undetermined in 15 (17.1%) patients with PCG. Figure 2 outlines the distribution of the anomalies associated with SCG-O, SCG-S, and SCG-A. We could obtain angle data for 56 eyes with SCG-A, of which 44 (78.6%) and 12 (21.4%) eyes had open and closed angles, respectively.

SCG-C was mostly observed following a surgery for a congenital idiopathic cataract (n=19, 57.6%), followed by congenital cataract associated with ocular anomalies or systemic diseases (n=7, 21.2%), and acquired cataract (n=3, 9.1%). There were four patients (12.1%) that the type of cataract could not be specified.

**Figure 3.**
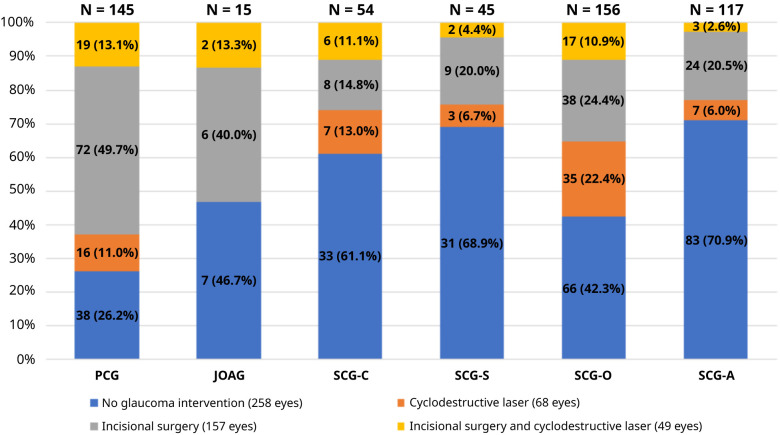
Type of glaucoma interventions in eyes with a glaucoma diagnosis. PCG, primary congenital glaucoma; JOAG, juvenile open angle glaucoma; SCG-C, secondary glaucoma following cataract surgery; SCG-S, secondary glaucoma associated with non-acquired systemic condition; SCG-O, secondary glaucoma associated with ocular anomalies; SCG-A, secondary glaucoma associated with acquired conditions.

While 157 glaucomatous eyes (29.5%) underwent an incisional surgery, 68 eyes (12.8%) underwent a cyclodestructive laser surgery. In contrast, 49 (9.2%) eyes required both incisional and cyclodestructive procedures at any time point during the follow-up period. Figure 3 presents the frequency of glaucoma intervention in each glaucoma type.

SCG-A had the highest proportion of favourable VA at the initial (57.5%) and latest (53.4%) visits. However, JOAG had the highest proportion of unfavourable VA at the initial (53.9%) and latest (73.3%) visits. We observed a higher proportion of unfavourable VA at the latest visit in the overall glaucoma cases (P=0.03), compared to that at the initial visit. A worsening of the VA was primarily observed in the SCG-O group. Despite an approach in the shift, it failed to attain a statistical significance (P=0.07). Moreover, the average IOP was 19.1±10.8 mmHg at the latest visit. All glaucoma types had significantly lower IOPs, compared to the baseline values (all p<0.001). Table 3 shows a comparison of the VA and IOP between the initial and latest visits for each glaucoma type.

**Table 3.** Visual acuity and intraocular pressure in eyes with a glaucoma diagnosis.

	Follow up period (months) median (IQR)	Initial visual acuity N (%)			Last visual acuity N (%)			P value *	Initial intraocular pressure (mmHg) mean (SE)	Last intraocular pressure (mmHg) mean (SE)	P value †
		Favorable	Moderately favorable	Unfavorable	Favorable	Moderately favorable	Unfavorable				
All glaucoma	60 (27 to 122)	103 (39.0%)	58 (22.0%)	103 (39.0%)	140 (34.7%)	64 (15.8%)	200 (49.5%)	0.03	28.5 (11.2)	19.1 (10.8)	<0.001
PCG	83 (36 to 134)	14 (24.6%)	14 (24.6%)	29 (50.9%)	30 (28.9%)	16 (15.4%)	58 (55.8%)	0.46	28.5 (11.0)	18.5 (12.3)	<0.001
JOAG	96 (15 to 156)	4 (30.8%)	2 (15.4%)	7 (53.9%)	3 (20.0%)	1 (6.7%)	11 (73.3%)	0.37	25.6 (14.3)	15.00 (6.8)	<0.001
SCG-C	47.5 (25 to 114)	12 (28.6%)	17 (40.5%)	13 (31.0%)	17 (34.0%)	18 (36.0%)	15 (30.0%)	0.25	27.2 (9.3)	17.2 (6.6)	<0.001
SCG-S	57 (27 to 111)	6 (30.0%)	10 (50.0%)	4 (20.0%)	13 (46.4%)	7 (25.0%)	8 (28.6%)	0.17	26.3 (9.5)	15.9 (7.6)	<0.001
SCG-O	75 (40 to 138)	17 (37.8%)	7 (15.6%)	21 (46.7%)	22 (21.2%)	16 (15.4%)	66 (63.5%)	0.07	29.9 (12.1)	21.6 (10.8)	<0.001
SCG-A	38 (16 to 81)	50 (57.5%)	8 (9.2%)	29 (33.3%)	55 (53.4%)	6 (5.8%)	42 (40.8%)	0.40	28.6 (11.1)	19.3 (11.3)	<0.001

* Calculated from the paired data of eyes with available initial visual acuity and latest visual acuity- all glaucoma 253 pairs, PCG 53 pairs, JOAG 13 pairs, SCG-C 42 pairs, SCG-S 17, SCG-O 41 pairs, and SCG-A 87 pairs. † Calculated from the paired data of eyes with available initial intraocular pressure and latest intraocular pressure- all glaucoma 634 pairs, PCG 129 pairs, JOAG 14 pairs, SCG-C 53 pairs, SCG-S 41, SCG-O 139 pairs, and SCG-A 110 pairs. PCG, primary congenital glaucoma; JOAG, juvenile open angle glaucoma; SCG-C, secondary glaucoma following cataract surgery; SCG-S, secondary glaucoma associated with non-acquired systemic condition; SCG-O, secondary glaucoma associated with ocular anomalies; SCG-A, secondary glaucoma associated with acquired conditions.

## Conclusions/discussion

Childhood glaucoma comprises a group of eye disorders that affect children from their birth with a juvenile onset. Our study had an average follow-up of 6 years. SCG-O (22.9%) was the most common subtype, followed by PCG (20.8%) and SCG-A (18.9%). The condition mostly affected boys, with the majority being bilateral cases, similar to previously published results. Primary glaucoma, both PCG and JOAG, and SCG-O commonly require at least one type of glaucoma intervention. Following the treatment, we observed significant IOP improvements in all subtypes. Nonetheless, half of the cases demonstrated unfavourable VA at the final visit.

Existing literature has reported on a varied distribution of the subtypes ^2, 8– 10, 12^. Most researchers have found a higher prevalence of secondary glaucoma, compared to primary glaucoma. However, reports from Canada ^6^, Egypt ^9^, Great Britain and the Republic of Ireland ^2^, and China ^7^ have found that the majority of cases comprised PCG. The prevalence of secondary glaucoma depends on the prevalence of its aetiology (e.g. childhood cataract, hereditary systemic disease), which can differ among regions and ethnicities. Furthermore, this variation can be explained by the diagnostic criteria and study design. The diagnostic criteria of the studies conducted before 2013 were not based on the CGRN classification. In addition, hospital-based studies tend to have a higher proportion of complex cases, such as patients with syndromic and systemic involvement than those conducted in population-based settings. Table 4 summarises the distribution of childhood glaucoma according to the CGRN classification ^1, 2, 5– 7, 9– 12^.

**Table 4.** Distribution of childhood glaucoma according to the Childhood Glaucoma Research Network classification.

Study	Number of glaucoma patients	Population	PCG	JOAG	SCG-C	SCG-S	SCG-O	SCG-A
**Hospital-based setting**								
Current study	338	2 Tertiary paediatric glaucoma clinics, Thailand	88 (26%)	8 (2.4%)	33 (9.8%)	32 (9.5%)	97 (28.7%)	80 (23.6%)
Senthil *et al.* 2019	275	Tertiary eye care, India	107(38.9%)	38 (13.8%)	22 (8%)	16 (5.8%)	48 (17.5%)	44 (16%)
Mokbel *et al.* 2018	207	Chief referral center, Egypt	114 (55%)	2 (1%)	15 (7.2%)	4 (2%)	11 (5.3%)	61 (29.5%)
Hoguet *et al.* 2016	122	Tertiary childhood glaucoma clinic, USA	39 (32%)	9 (7.4%)	22 (18%)	14 (11.5%)	10 (8.2%)	28 (22.9%)
Fung *et al.* 2013	152 *	Dallas Glaucoma Registry, USA	46 (30.3%)	10 (6.6%)	30 (19.7%)	18 (11.8%)	16 (10.5%)	32 (21.1%)
Qiao *et al.* 2009 †	948	Hospitalized paediatric patients, Beijing, China	486 (51.3%)	63 (6.6%)	125 (13.2%)	40 (4.2%)	61 (6.4%)	173 (18.3%)
Taylor *et al.* 1999 †	296 *	Hospital for sick children, Toronto, Canada	117 (39.5%)	7 (2.4%)	61 (20.6%)	32 (10.8%)	38 (12.8%)	41 (13.9%)
Barsoum-Homsy *et al.* 1986	63	Paediatric glaucoma clinic, Montreal, Canada	14 (22.2%)	0	7 (11.1%)	9 (14.2%)	24 (38%)	9 (14.2%)
**Population-based setting**								
Aponte *et al.* 2010 †	30	Olmstead county residents, Minnesota, USA	1 (3.3%)	4 (13.3%)	6 (20%)	4 (13.4%)	2 (6.7%)	13 (43.3%)
Papadppoulos *et al.* 2007	91 *	British Ophthalmic Surveillance Unit, Great Britain and Republic of Ireland	45 (49.4%)	2 (2.2%)	16 (17.6%)	12 (13.2%)	6 (6.6%)	10 (11%)

* We excluded patients who were glaucoma suspect or had an unknown diagnosis. † The diagnosis was reclassified from the original articles to follow the CGRN classification. PCG, primary congenital glaucoma; JOAG, juvenile open angle glaucoma; SCG-C, secondary glaucoma following cataract surgery; SCG-S, secondary glaucoma associated with non-acquired systemic condition; SCG-O, secondary glaucoma associated with ocular anomalies; SCG-A, secondary glaucoma associated with acquired conditions

Leukocoria or corneal haze were the leading presenting symptom in PCG (46.9%) and SCG-O (77%) similar with previous studies ^16– 18^. Moreover, most patients with JOAG were diagnosed without any symptoms. This could partially explain the most advanced stage at the time of diagnosis and the highest proportion of unfavourable VA outcomes in the aforementioned subtype. High IOP was the leading clue for glaucoma diagnosis. Furthermore, an enlarged corneal diameter was considered an important sign of PCG ^16^.

We detected a family history of glaucoma in 6.4% of the 234 patients with an available family history. A study by Fung *et al.* reported on a family history of glaucoma in 17% patients with paediatric GS ^5^. This high rate could be attributed to the tendency of having the eyes checked because of a family history of glaucoma. In our study, an exclusion of the GS cases would have reduced the rate of positive family history from 6.4% to only 5.7% (10 out of 174 patients). This value was half of that reported by Papadopoulos *et al.* (11%) ^2^. Despite the association of PCG and JOAG with certain mutations ^19^, our cohort revealed a positive family history in none of the PCG cases and in only one JOAG case. Therefore, the PCG cases in Thailand were sporadic rather than inherited.

In line with the published literature, we found that surgical interventions were mostly required in the primary type of glaucoma, both PCG and JOAG. Moreover, medication was the mainstay of treatment for most secondary glaucoma cases ^2^. In addition, SCG-O cases reported a high rate of surgical intervention. The pathology of the above-mentioned subtype is related to angle dysgenesis, which usually makes it difficult to control the disease.

Ramkrishanan *et al*. reported a significant improvement of VA, which was sustained for at least four years of follow-up ^20^. This was in contrast to the marginally significant worsening of VA observed in our study. This disparity could be attributed to a greater proportion of PCG cases in the study conducted by Ramkrishanan *et al.* The improvement of VA in their study was attributed to an improved corneal clarity following surgery.

We found that the SCG-A cases had the most favourable VA at the latest visit and the best initial VA. The majority of the cases included steroid-induced glaucoma, which might be more controllable than the subtypes related to congenital ocular malformations, such as PCG and SCG-O. However, we found an overall worse VA, compared to that reported in previous publications ^6, 11, 20^. This discrepancy could be explained by the following aspects. First, we documented a high proportion of unfavourable VA during the initial visits. Khitri *et al.* reported on an association between poor vision at diagnosis and visual impairment (<20/200) ^21^. Second, our cases were diagnosed at an extremely young age, particularly in the PCG (median age 0.5 years) and SCG-O (median age 0.3 years) groups. Studies on the PCG subtype reported on final VA <20/200 in children diagnosed before the age of three months regardless of their IOP levels ^22, 23^. It was hypothesised that the earlier presentation reflected the poorer development of the angle. In other words, the disease was more severe. Nevertheless, the study design and definition of unfavourable VA differed among the studies.

Our study had the strength of being a large cohort study with a long follow-up duration. Our data also represents the majority of childhood glaucoma cases in Thailand. However, it had several limitations. First, there were some incomplete data because of the retrospective design. Moreover, information on some clinical examinations, such as VA and IOP could not always be obtained in children at every clinic visit. Second, the long follow-up period resulted in a shift in the IOP measurement methods from a handheld contact tonometer (Tono-Pen; Reichert, New York, USA) to a rebound tonometer (iCare TAO1i, Tiolat Oy, Helsinki, Finland) in extremely young or non-cooperative children. Third, there was inadequate information to clearly identify the cause of unfavourable VA outcomes. Future research should explore this underlying issue.

In conclusion, data from the referral centres in Thailand showed a higher prevalence of secondary glaucoma than primary glaucoma. Using the CGRN classification, secondary glaucoma associated with non-acquired ocular anomalies was found to be the most common subtype. All subtypes, including primary glaucoma, were sporadic. A majority of the cases had unfavourable visual outcomes. These real-world findings are fundamental data and provide a better understanding of childhood glaucoma.

## Data availability

### Underlying data

Harvard Dataverse: Childhood glaucoma, https://doi.org/10.7910/DVN/V3HFNF ^24^.

Data are available under the terms of the Creative Commons Zero "No rights reserved" data waiver (CC0 1.0 Public domain dedication).
