# Supplementary material for: Characteristics and long-term outcomes of childhood glaucoma: a retrospective-cohort study
Source: F1000Res. 2022 Jan 5;10:165. Originally published 2021 Mar 1. [Version 2] doi: 10.12688/f1000research.51256.2 (PMC8738969; doi:10.12688/f1000research.51256.2)
Supplement: Peer review history

# Peer review: Reviewer response for version 2

**DOI:** 10.5256/f1000research.84929.r118762

**Published:** 2022-01-07

**Authors:** Chungkwon Yoo

**Affiliations:** 1 Department of Ophthalmology, Korea University College of Medicine, Seoul, South Korea

The authors dealt properly with the issues previously raised in their revised manuscript. 

 I have no further comments to make.

Is the work clearly and accurately presented and does it cite the current literature?

Yes

If applicable, is the statistical analysis and its interpretation appropriate?

Yes

Are all the source data underlying the results available to ensure full reproducibility?

Yes

Is the study design appropriate and is the work technically sound?

Yes

Are the conclusions drawn adequately supported by the results?

Partly

Are sufficient details of methods and analysis provided to allow replication by others?

Yes

Reviewer Expertise:

glaucoma surgery

I confirm that I have read this submission and believe that I have an appropriate level of expertise to confirm that it is of an acceptable scientific standard.

# Peer review: Reviewer response for version 2

**DOI:** 10.5256/f1000research.84929.r118761

**Published:** 2022-01-06

**Authors:** Kazuhiko Mori, Hiroki Mieno

**Affiliations:** 1 Department of Ophthalmology, Kyoto Prefectural University of Medicine, Kyoto, Japan

The author provided the appropriate responses to all of the reviewer's comments.

 No further comments to make.

Is the work clearly and accurately presented and does it cite the current literature?

Yes

If applicable, is the statistical analysis and its interpretation appropriate?

Yes

Are all the source data underlying the results available to ensure full reproducibility?

Yes

Is the study design appropriate and is the work technically sound?

Yes

Are the conclusions drawn adequately supported by the results?

Partly

Are sufficient details of methods and analysis provided to allow replication by others?

Yes

Reviewer Expertise:

glaucoma

We confirm that we have read this submission and believe that we have an appropriate level of expertise to confirm that it is of an acceptable scientific standard.

# Peer review: Reviewer response for version 1

**DOI:** 10.5256/f1000research.54403.r80446

**Published:** 2021-04-01

**Authors:** Chengguo Zuo

**Affiliations:** 1 State Key Laboratory of Ophthalmology, Zhongshan Ophthalmic Center, Guangzhou, China

This interesting study analyzed the clinical characteristics and treatment results of patients with childhood glaucoma who had visited the glaucoma clinics at the Queen Sirikit National Institute of Child Health and the King Chulalongkorn Memorial Hospital within 10 years, and provided effective data and analysis for a better understanding of children’s glaucoma in Thailand. There are several questions that need to be answered:

1. In the "Results" of the "Abstract", “Most patients presented with a high initial intraocular pressure (IOP) of 28.5±11.2 mmHg.” Does the patients here refer to all patients or just for the patients with a high initial IOP?
2. In the “Results”, you described that “The average age at presentation was 3.91±4.40 years (median 1.75: interquartile range, 0.25–6.75 years).” But in the “Table 1”, the average age at presentation of the total patients is 1.6(0.3 to 6.8) years. Please give a more detailed explanation here or check data again.
3. In this study, the authors only collected the family history of 234 of the 423 patients, as you described that “We detected a family history of glaucoma in 6.4% of the 234 patients with an available family history.” in the “Conclusions/discussion”. We suggest that the description of this in the previous part “Result” that “We recorded a family history of glaucoma in 15 (6.4%) patients” need to be added the information that the data was from 234 patients, so as not to cause misleading or doubt. Besides, when analyzing the family history of patients, the author did not analyze the fact that the family history of not all the patients had been collected. This will inevitably lead to a certain degree of bias. Without analyzing this, the conclusion that” Therefore, the PCG cases in Thailand were sporadic rather than inherited.” could be not that reliable.
4. There are several places in the article that need to be supported by the literature: a）：In the “Conclusion” that “The condition mostly affected boys, with the majority being bilateral cases, similar to previously published results.”b）：In the “Conclusion” that “In addition, SCG-O cases reported a high rate of surgical intervention.”
5. In the “Conclusion”, you described that “This disparity could be attributed to a greater proportion of PCG cases in the study conducted by Ramkrishanan *et al*.” Please Please provide the PCG ratio of this research and the comparison with that of your study to compare the difference between the two more intuitively.
6. In the description SCG-A had the most favourable VA at the latest visit and the best initial VA, you described that “The majority of the cases included steroid-induced glaucoma, which might be more controllable than the subtypes related to congenital ocular malformations, such as PCG and SCG-O.” But from Figure 2, steroid-induced glaucoma accounts for 29%, which is not so high. Does the authors mean “The majority of the cases such as steroid-induced glaucoma, which might be more controllable than the subtypes related to congenital ocular malformations…”?

Is the work clearly and accurately presented and does it cite the current literature?

Yes

If applicable, is the statistical analysis and its interpretation appropriate?

Partly

Are all the source data underlying the results available to ensure full reproducibility?

Yes

Is the study design appropriate and is the work technically sound?

Yes

Are the conclusions drawn adequately supported by the results?

Yes

Are sufficient details of methods and analysis provided to allow replication by others?

Yes

Reviewer Expertise:

Glaucoma

I confirm that I have read this submission and believe that I have an appropriate level of expertise to confirm that it is of an acceptable scientific standard.

# Response

**Published:** 2021-12-14

**Authors:** Sunee Chansangpetch

**Affiliations:** Chulalongkorn University, Thailand

This interesting study analyzed the clinical characteristics and treatment results of patients with childhood glaucoma who had visited the glaucoma clinics at the Queen Sirikit National Institute of Child Health and the King Chulalongkorn Memorial Hospital within 10 years, and provided effective data and analysis for a better understanding of children’s glaucoma in Thailand. There are several questions that need to be answered: 

 1. In the "Results" of the "Abstract", “Most patients presented with a high initial intraocular pressure (IOP) of 28.5±11.2 mmHg.” Does the patients here refer to all patients or just for the patients with a high initial IOP?

**Response: **We apologize that the sentence was not clear. The IOP of 28.5±11.2 mmHg referred to all patients’ IOP. We have modified the sentence as follows:

 Abstract: “Most patients presented with a high initial intraocular pressure (IOP). The average initial IOP of all patients was 28.5±11.2 mmHg.”

 2. In the “Results”, you described that “The average age at presentation was 3.91±4.40 years (median 1.75: interquartile range, 0.25–6.75 years).” But in the “Table 1”, the average age at presentation of the total patients is 1.6(0.3 to 6.8) years. Please give a more detailed explanation here or check data again.

**Response: **Thank you very much for catching this. The median age at presentation was 1.58. We have corrected the error in the Results:

 Results: “The average age at presentation was 3.91±4.40 years (median 1.58: interquartile range, 0.25–6.75 years).”

 3. In this study, the authors only collected the family history of 234 of the 423 patients, as you described that “We detected a family history of glaucoma in 6.4% of the 234 patients with an available family history.” in the “Conclusions/discussion”. We suggest that the description of this in the previous part “Result” that “We recorded a family history of glaucoma in 15 (6.4%) patients” need to be added the information that the data was from 234 patients, so as not to cause misleading or doubt. Besides, when analyzing the family history of patients, the author did not analyze the fact that the family history of not all the patients had been collected. This will inevitably lead to a certain degree of bias. Without analyzing this, the conclusion that” Therefore, the PCG cases in Thailand were sporadic rather than inherited.” could be not that reliable.

**Response:** We agree with the reviewer’s comment. We have added that the family history data was obtained from 234 patients and removed the conclusion of the sporadic nature in the Abstract to avoid misleading. The Conclusions/Discussion section have also been modified.

 Abstract - results: We recorded a family history of glaucoma in 6.4% of patients of the 234 patients with an available family history.

 Conclusions/Discussion - 4 ^th^ paragraph: “The true frequency of familial glaucoma, however, may be higher, as the data were available for only 55% of our patients. In addition, getting a family history without examining each family member tends to underestimate the actual occurrence of glaucoma in the family.”

 4. There are several places in the article that need to be supported by the literature: a）：In the “Conclusion” that “The condition mostly affected boys, with the majority being bilateral cases, similar to previously published results.”b）：In the “Conclusion” that “In addition, SCG-O cases reported a high rate of surgical intervention.”

**Response: **Thank you for the comment. The following references have been added.

 To (a): 16. American Academy of Ophthalmology. Glaucoma in Children and Adolescents. In: Tanna AP, Lin SC, Boland MV, et al., eds. *Basic and Clinical Science Course Section 10 Glaucoma.* San Francisco: American Academy of Ophthalmology; 2020.

 To (b): 5. Fung DS, Roensch MA, Kooner KS, Cavanagh HD, Whitson JT. Epidemiology and characteristics of childhood glaucoma: results from the Dallas Glaucoma Registry. *Clinical Ophthalmology. *2013;7:1739-1746.

 5. In the “Conclusion”, you described that “This disparity could be attributed to a greater proportion of PCG cases in the study conducted by Ramkrishanan *et al*.” Please Please provide the PCG ratio of this research and the comparison with that of your study to compare the difference between the two more intuitively.

**Response:** We appreciate the reviewer’s comment. We have included the proportion of Ramkrishanan’s study in comparison with our study.

 Conclusions/Discussion - 7 ^th^ paragraph: “This disparity could be attributed to a greater proportion of PCG cases in the study conducted by Ramkrishanan *et al.* (Ramkrishanan *et al*. 73.3% vs our study 20.8%).”

 6. In the description SCG-A had the most favourable VA at the latest visit and the best initial VA, you described that “The majority of the cases included steroid-induced glaucoma, which might be more controllable than the subtypes related to congenital ocular malformations, such as PCG and SCG-O.” But from Figure 2, steroid-induced glaucoma accounts for 29%, which is not so high. Does the authors mean “The majority of the cases such as steroid-induced glaucoma, which might be more controllable than the subtypes related to congenital ocular malformations…”?

**Response:** Thank you. The sentence has been revised as suggested.

 Conclusions/Discussion - 8 ^th^ paragraph: “The majority of the cases included steroid-induced glaucoma, trauma and uveitis. In general, acquired conditions might be more controllable than the subtypes related to congenital ocular malformations, such as PCG and SCG-O.”

# Peer review: Reviewer response for version 1

**DOI:** 10.5256/f1000research.54403.r80450

**Published:** 2021-04-01

**Authors:** Kazuhiko Mori, Hiroki Mieno

**Affiliations:** 1 Department of Ophthalmology, Kyoto Prefectural University of Medicine, Kyoto, Japan

This article successfully reports the clinical characteristics and treatment outcomes of patients with childhood glaucoma from the real-world large data of 10 years in Thailand.

 The statistics used are appropriate and the interpretations are consistent. Overall findings in this study are important to the field. However, there are a few points need to be fixed, and discussion needs more work to improve the interpretation of the results.

**Major comments:**

1. The discussion about the reason of unfavorable visual outcome is insufficient. Authors should dig much deeper into the reason of visual impairment of their patients, whether it was really unavoidable or not.
  For example, JOAG group mainly seems to have clear cornea where disc is visible (14/15) and good IOP control (25.6 to 15.0 mmHg), however, the unfavorable cases were increasing compared to the initial VA. What caused the unwilling visual outcome?
  Is it due to the corneal haziness or glaucoma progression?  Inadequate orthoptic exercises? Or merely gathering of very severe cases to the tertiary hospitals?
  If there are any possible clues or suggestions to improve, it might be a good help for the readers who are struggling under the same situation.
2.  As the authors mentioned, surgical interventions were mostly required in the primary type of glaucoma, both PCG and JOAG, and especially SCG-O. However, from this study, no glaucoma intervention was performed to 46.7, and 42.3% of the patients of JOAG and SCG-O, respectively. Explain the reason why almost a half of the patients did not need the surgical intervention.

**Minor comments:**

1. Need the details of surgical procedures.
2. Are there any relations between the laterality and visual outcome?

Is the work clearly and accurately presented and does it cite the current literature?

Yes

If applicable, is the statistical analysis and its interpretation appropriate?

Yes

Are all the source data underlying the results available to ensure full reproducibility?

Yes

Is the study design appropriate and is the work technically sound?

Yes

Are the conclusions drawn adequately supported by the results?

Partly

Are sufficient details of methods and analysis provided to allow replication by others?

Yes

Reviewer Expertise:

glaucoma

We confirm that we have read this submission and believe that we have an appropriate level of expertise to state that we do not consider it to be of an acceptable scientific standard, for reasons outlined above.

# Response

**Published:** 2021-12-14

**Authors:** Sunee Chansangpetch

**Affiliations:** Chulalongkorn University, Thailand

This article successfully reports the clinical characteristics and treatment outcomes of patients with childhood glaucoma from the real-world large data of 10 years in Thailand.

 The statistics used are appropriate and the interpretations are consistent. Overall findings in this study are important to the field. However, there are a few points need to be fixed, and discussion needs more work to improve the interpretation of the results.

**Response: We appreciate the reviewer's comment.**

 Major comments:

 1. The discussion about the reason of unfavorable visual outcome is insufficient. Authors should dig much deeper into the reason of visual impairment of their patients, whether it was really unavoidable or not.

 For example, JOAG group mainly seems to have clear cornea where disc is visible (14/15) and good IOP control (25.6 to 15.0 mmHg), however, the unfavorable cases were increasing compared to the initial VA. What caused the unwilling visual outcome?

 Is it due to the corneal haziness or glaucoma progression?  Inadequate orthoptic exercises? Or merely gathering of very severe cases to the tertiary hospitals?

 If there are any possible clues or suggestions to improve, it might be a good help for the readers who are struggling under the same situation.

**Response: Thank you for bringing up this important issue. As also mentioned by another reviewer, we agree that the factors associated with visual outcome would be worth exploring. The ordinal regression analysis showed that less favorable of initial visual acuity (VA) and high last IOP were significantly associated with poor visual outcome. In comparison to SCG-C, PCG, JOAG and SCG-O diagnoses were significantly associated with poor visual outcome. No significant risk of poor visual outcome was identified for SCG-S and SCG-A when compared to SCG-C.**

**As all patients were primarily seen by pediatric ophthalmologists (please refer to the response to reviewer 1’s query No.3), we believe that inadequate orthoptic exercise and inappropriate refractive correction would be less the case. However, we could not assess the adherence to the treatment and follow-up with the pediatric ophthalmologists.**

**To elaborate more on the progression to unfavorable visual impairment, we further investigated the change of the VA group. Table 6 in the revised manuscript showed that most patients had no change in the VA group between the initial VA and the last VA. Most eyes with unfavorable last VA had already presented with unfavorable VA. The SCG-C tended to have an improvement of the VA. On the other hand, SCG-O had the highest proportion of worsening VA. There was no improvement in the VA group in any of the JOAG.**

**There were 12 eyes of SCG-O that showed worsening of the VA to the unfavourable VA at the latest visit. Almost all cases had limited visual potential due to cornea and/or anterior segment anomalies (Peters anomaly, microcornea and sclerocornea). These patients had their initial visit at a young age (all < 6 years) and the initial VA was recorded as ‘fix and follow’, which was classified in this study as ‘favourable VA’. However, the final VA ranged from HM to FC 3 ft, which could be explained by their cornea and/or anterior segment conditions. There were 2 cases of congenital ectopia lentis. One of them underwent cataract surgery without IOL implantation. The other developed retinal detachment after trabeculectomy. Both had the final VA of hand motion.**

**Among the 13 JOAG with available initial visual acuity and latest visual acuity, there were 2 eyes that showed worsening of the VA group. The first case presented with a C:D ratio of 0.9 in the right eye. The contralateral eye had no light perception at the presentation. The patient had successful trabeculectomy in the right eye but lost to follow-up afterwards. She came back with the vision of PL and full disc cupping. The second case had an initial C:D ratio of 0.95 and an initial VA of 20/100. Although the patient responded well to the treatment and the IOPs were in the low teens, the VA slowly got worse. After approximately 12 years, the last VA was hand motion. Regarding 7 eyes with unfavourable final VA and unfavourable initial VA, all C:D ratios at presentation were at least 0.9.**

**Our data suggested that the initial VA and glaucoma stage at presentation were the main factors determining the unfavourable VA outcome. The underlying ocular abnormalities tended to limit the visual potential in SCG-O, the anterior segment dysgenesis in particular. We now mentioned this point in the 9 ^th^ and 10 ^th^ paragraph of the Discussion.**

 2. As the authors mentioned, surgical interventions were mostly required in the primary type of glaucoma, both PCG and JOAG, and especially SCG-O. However, from this study, no glaucoma intervention was performed to 46.7, and 42.3% of the patients of JOAG and SCG-O, respectively. Explain the reason why almost a half of the patients did not need the surgical intervention.

**Response: We thank the reviewer for the comment. Although PCG, JOAG and SCG-O had the high rate of surgical intervention, a high proportion of eyes that had no glaucoma interventions was observed in our study. The primary reason for not receiving surgical intervention was that the IOP could be controlled with medications. All patients with evidence of high IOP were offered surgical intervention (trabeculotomy and goniotomy) for PCG. The option of surgical intervention was discussed with the families of those who had previously been treated with medication. After seeing the well-controlled IOP, some parents preferred to continue with the medication. Unlike PCG, the surgical intervention was not necessarily offered to all SCG-O and JOAG patients. The decision to perform surgery in SCG-O was mainly based on the IOP and visual prognosis. Conservative treatment with medications was preferred If there was very limited vision potential such as nystagmus or visual acuity of light perception. For JOAG, the approach was quite similar to that for adult glaucoma. The majority of cases that did not receive glaucoma intervention underwent selective laser trabeculoplasty and were able to achieve target IOPs, though with medications.**

**We have now explained these reasons in the 6 ^th^ paragraph Discussion. Additional information of the patients that had no glaucoma intervention was listed below.**

**PCG: 38 eyes from 21 patients**

-
  - **9 eyes (5 patients) were lost to follow-up.**
  - **2 eyes (2 patients) underwent evisceration due to severe buphthalmos. (One eye also had the ruptured globe.)**
  - **1 eye (1 patient) had severe corneal ulcer and became phthisis.**
  - **2 eyes (1 patient) developed late-onset infantile cataract. After lens aspiration the IOP went down to mid teen with single medication.**
  - **16 eyes (8 patients) had the presenting IOP of less than 20 mmHg because they were treated with medications before the referral. The parents prefer to continue with the medication.**
  - **6 eyes (3 patients) had high presenting IOP and were scheduled for the surgery. After initiating the medication during, the IOP went down to mid teen with 2-3 medications and the parents refused to have the surgery.**
  - **2 eyes (1 patients) had no specified reason in the medical record.**

** SCG-O: 66 eyes from 49 patients**

-
  - **4 eyes (4 patients) were lost to follow-up.**
  - **1 eye (1 patient) underwent evisceration due to impending ruptured descematocele.**
  - **30 eyes (17 patients) had the presenting IOP of less than 20 mmHg because they were treated with medications before the referral.**
  - **8 eyes (5 patients) had high presenting IOP. After initiating the med, the IOP went down to mid teen with 2-3 medications.**
  - **20 eyes (15 patients) had initial poor visual prognosis, inoperable condition (e.g. marked thin sclera) or contraindication for surgery (e.g. underlying heart disease)**
  - **3 eyes (3 patients) had no specified reason in the medical record.**

** JOAG: 7 eyes from 4 patients**

-
  - **2 eyes (1 patient) were lost to follow-up.**
  - **3 eyes (2 patients) received selective laser trabeculoplasty.**
  - **2 eyes (1 patient) had IOP lowered to teen by medications.**

Minor comments:

 1. Need the details of surgical procedures.

**Response: Thank you very much. We have now included a table that summarizes the type of surgical procedures for each glaucoma subtype. (new Table 3) Please note that some eyes might require more than 1 surgery, thus the overall frequency in this table may differ from that showed in Figure 3.**

 2. Are there any relations between the laterality and visual outcome?

**Response: Thank you for the comment. The statistical analysis with chi-square test showed no association between the laterality and visual outcome (p=0.62).**

# Peer review: Reviewer response for version 1

**DOI:** 10.5256/f1000research.54403.r80447

**Published:** 2021-03-15

**Authors:** Chungkwon Yoo

**Affiliations:** 1 Department of Ophthalmology, Korea University College of Medicine, Seoul, South Korea

The authors evaluated the clinical characteristics and treatment outcomes of patients with childhood glaucoma seen in their glaucoma clinics for 10 years. This is a clinically interesting study. However, there are some issues which need to be addressed.  

1. Please add the specific information on the surgical interventions used and their proportions in each glaucoma subtype: trabeculectomy, goniotomy, trabeculotomy or tube surgery?
2. Corneal diameters at initial visit?
3. Poor VA outcome may have resulted from factors other than glaucoma..e.g. inadequately corrected VA or coexisting eye problems. How many of the study eyes had been seen by pediatric ophthalmologists?
4. You may analyze the factors associated with poor visual outcome.
5. Given the fact that PCG needs surgical management, the proportion of nonsurgically managed eyes among PCG cases was unexpectedly high. What were the reasons for nonsurgical management in those patients? Please address this issue in the discussion section.
6. The authors concluded that all subtypes of glaucoma were sporadic. Although the patient-reported or family-reported 'family history of glaucoma' was uncommon, the actual familial occurrence may be more than can be assessed by history taking. Unless each family member was examined for glaucoma, you may not be certain that all subtypes of childhood glaucoma were sporadic.

Is the work clearly and accurately presented and does it cite the current literature?

Yes

If applicable, is the statistical analysis and its interpretation appropriate?

Yes

Are all the source data underlying the results available to ensure full reproducibility?

Yes

Is the study design appropriate and is the work technically sound?

Yes

Are the conclusions drawn adequately supported by the results?

Partly

Are sufficient details of methods and analysis provided to allow replication by others?

Yes

Reviewer Expertise:

glaucoma surgery

I confirm that I have read this submission and believe that I have an appropriate level of expertise to state that I do not consider it to be of an acceptable scientific standard, for reasons outlined above.

# Response

**Published:** 2021-12-14

**Authors:** Sunee Chansangpetch

**Affiliations:** Chulalongkorn University, Thailand

The authors evaluated the clinical characteristics and treatment outcomes of patients with childhood glaucoma seen in their glaucoma clinics for 10 years. This is a clinically interesting study. However, there are some issues which need to be addressed.   

 1. Please add the specific information on the surgical interventions used and their proportions in each glaucoma subtype: trabeculectomy, goniotomy, trabeculotomy or tube surgery?Thank you very much. The table below summarizes the type of surgical procedures for each glaucoma subtype. Please note that some eyes might require more than 1 surgery, thus the overall frequency in this table may differ from the frequency shown in Figure 3.

**Response: Thank you very much. We have added a table that summarizes the type of surgical procedures for each glaucoma subtype in the revised version of the manuscript. (new Table 3) Please note that some eyes might require more than 1 surgery, thus the overall frequency in this table may differ from the frequency shown in Figure 3.**

 2. Corneal diameters at initial visit?

**Response: We apologize for the unclear description. The Refraction, Corneal diameter, Cup to disc ratio listed in Table 1 were the findings from initial visits. We have now updated the table’s footnote.**

**Table 1 - footnote: “Refraction, corneal diameter, and cup to disc ratio were the measurements taken at first visit.”**

 3. ****Poor VA outcome may have resulted from factors other than glaucoma..e.g. inadequately corrected VA or coexisting eye problems. How many of the study eyes had been seen by pediatric ophthalmologists?

**Response: We absolutely agree that several factors contribute to poor VA outcome. At the Queen Sirikit National Institute of Child Health (QSNICH), all children are primarily seen by QSNICH’s pediatric ophthalmologists. The patients are sent to pediatric glaucoma team for glaucoma consultation. They were still followed by pediatric ophthalmologists for other conditions such as refractive error, cataract and other coexisting eye problems. All surgical interventions except for glaucoma procedures are performed by the QSNICH’s pediatric ophthalmologists. However, due to the limitations of a retrospective study, it would be difficult to clearly delineate the cause of poor visual outcome in each patient. We also acknowledge this issue in our Discussion.**

**Conclusions/Discussion - 10 ^th^ paragraph: “It should be noted that unfavourable VA could be a result from a combination of factors other than glaucoma such as underlying ocular pathology, uncorrected refractive error or amblyopia. As all patients were concomitantly seen by pediatric ophthalmologists, we believe that inadequate orthoptic exercise and inappropriate refractive correction would be less the case. However, due to the limitations of a retrospective study, it would be difficult to clearly delineate the cause of poor visual outcome in each patient.”**

 4. You may analyze the factors associated with poor visual outcome.

**Response: Thank you for the astute comment. As suggested by the reviewer, we performed the analysis with an ordinal logistic regression to explore the factors associated with visual outcome. The complete case analysis of 219 eyes showed that less favorable of initial visual acuity and high last IOP were significantly associated with poor visual outcome. In comparison to SCG-C, PCG, JOAG and SCG-O diagnoses were significantly associated with poor visual outcome. No significant risk of poor visual outcome was identified for the SCG-S and SCG-A groups when compared to the SCG-C group. We have updated the Methods and Results sections to include the description of the statistical analysis and the table in the revised manuscript. (new Table 5).**

 5. Given the fact that PCG needs surgical management, the proportion of nonsurgically managed eyes among PCG cases was unexpectedly high. What were the reasons for nonsurgical management in those patients? Please address this issue in the discussion section.

**Response: Thank you for the comment. The primary reason for not receiving surgical intervention was that the IOP could be controlled with medications. All patients with evidence of high IOP were offered surgical intervention (trabeculotomy and goniotomy) for PCG. The option of surgical intervention was discussed with the families of those who had previously been treated with medication. After seeing the well-controlled IOP, some parents preferred to continue with the medication.**

**For additional information of the patients who did not have glaucoma intervention, please refer to the response to reviewer 2’s query No.2. We have now included this explanation in the 6 ^th^ paragraph of the Discussion.**

 6. The authors concluded that all subtypes of glaucoma were sporadic. Although the patient-reported or family-reported 'family history of glaucoma' was uncommon, the actual familial occurrence may be more than can be assessed by history taking. Unless each family member was examined for glaucoma, you may not be certain that all subtypes of childhood glaucoma were sporadic.

**Response: We thank the reviewer for the insightful comment and agree with the suggestion. We have removed the conclusion in the Abstract regarding this issue and have modified the Discussion to avoid misleading.**

**Conclusions/Discussion - 4 ^th^ paragraph: “The true frequency of familial glaucoma, however, may be higher, as the data were available for only 55% of our patients. In addition, getting a family history without examining each family member tends to underestimate the actual occurrence of glaucoma in the family.”**
